# Exhaled IL-8 in Systemic Lupus Erythematosus with and without Pulmonary Fibrosis

**DOI:** 10.1007/s00005-014-0270-5

**Published:** 2014-02-04

**Authors:** Agnieszka Nielepkowicz-Goździńska, Wojciech Fendler, Ewa Robak, Lilianna Kulczycka-Siennicka, Paweł Górski, Tadeusz Pietras, Ewa Brzeziańska, Adam Antczak

**Affiliations:** 1 Department of General and Oncological Pneumology, Medical University of Lodz, Kopcińskiego 22, 90-153 Lodz, Poland; 2 Department of Pediatrics, Oncology, Hematology and Diabetology, Medical University of Lodz, Lodz, Poland; 3 Department of Dermatology and Venereology, Medical University of Lodz, Lodz, Poland; 4 Department of Pneumonology and Allergy, Medical University of Lodz, Lodz, Poland; 5 Department of Molecular Bases of Medicine, Medical University of Lodz, Lodz, Poland

**Keywords:** Systemic lupus erythematosus, Interleukin-8, Exhaled breath condensate, Bronchoalveolar lavage fluid, Pulmonary fibrosis

## Abstract

The purpose of this study is to evaluate the relationship between the concentration of interleukin-8 (IL-8) in exhaled breath condensate (EBC) and bronchoalveolar lavage fluid (BALF) with the disease activity score and pulmonary function of systemic lupus erythematosus (SLE) patients with and without pulmonary fibrosis. Thirty-four SLE patients and 31 healthy controls were enrolled and evaluated using high-resolution computed tomography (HRCT), pulmonary function tests, systemic lupus activity measure (SLAM), assessing BALF and EBC. IL-8 levels in BALF and EBC samples were measured with an enzyme-immunosorbent assay kit. The mean (±SEM) IL-8 concentrations in BALF and EBC were higher in SLE patients compared to healthy controls (34.84 ± 95.0 vs. 7.65 ± 21.22 pg/ml, *p* < 0.001; 3.82 ± 0.52 pg/m vs. 1.7 ± 1.7 pg/ml, *p* < 0.001, respectively). SLE patients had increased percentage of neutrophils in BALF when compared with control group (1.00 ± 5.99 vs. 0.00 ± 0.56 %, *p* = 0.0003). Pulmonary fibrosis in HRCT was found in 50 % of SLE patients. The disease activity scored by SLAM was significantly higher and total lung capacity was significantly lower in SLE patients with pulmonary fibrosis (8.00 ± 3.17 vs. 6.00 ± 2.31, *p* = 0.01; 88.00 ± 28.29 vs. 112.00 ± 21.08 % predicted, *p* = 0.01, respectively). In SLE patients with pulmonary fibrosis, correlations were found between SLAM and IL-8 concentration in BALF, forced expiratory volume in 1 s and forced vital capacity (*r* = 0.65, *p* = 0.006; *r* = −0.53, *p* = 0.035; *r* = −0.67, *p* = 0.006, respectively). Our results indicate that IL-8 plays an important role in the pathogenesis of SLE. An increased concentration of IL-8 according to BALF could be considered as a useful biomarker of SLE activity and pulmonary fibrosis in SLE.

## Introduction

Cytokines play an important role in the process of organ damage in systemic lupus erythematosus (SLE). An increased level of cytokines was found in the serum, urine and cerebrospinal fluid of SLE patients and the concentration of these biomarkers was associated with the severity of the disease (Aringer and Smolen 2004; Dean et al. 2000). Interleukin-8 (IL-8) is suggested to play an important role both in the pathogenesis of SLE and pulmonary fibrosis (Keane and Strieter 2002). This is the first study estimating concentration of IL-8 in exhaled breath condensate (EBC) and bronchoalveolar lavage fluid (BALF) of SLE patients with and without pulmonary fibrosis with the aim of better understanding the degree to which IL-8 can be used as a prognostic factor in SLE patients. We have previously reported increased IL-6 and IL-10 concentrations in the BALF and IL-10 concentration in EBC in the same group of SLE patients (Nielepkowicz-Goździńska et al. 2013).

## Materials and Methods

The study comprised 34 patients [fulfilling the revised American College of Rheumatology criteria for the diagnosis of SLE (Hochberg 1997)] recruited from outpatient clinics and 31 healthy controls (never-smokers members of the hospital staff and medical students with normal spirometry results) matched for sex and age. In all patients high-resolution computed tomography (HRCT), spirometry, body plethysmography, diffusion capacity for carbon monoxide corrected for hemoglobin concentration (DLCOc) measurement, EBC and BALF sample collection were performed. In all healthy individuals spirometry and EBC collection were performed, while BALF fluid collection was carried out in 20. Patients were divided into two groups, one with and one without pulmonary fibrosis in HRCT chest studies. The study was carried out with the approval of the Ethics Committee of the Medical University of Lodz (RNN/146/07/KE). A written informed consent was obtained from all patients.

Sixteen patients were taking corticosteroids (prednisone: 5–20 mg/day), three of them were treated with a combination of corticosteroids and azathioprine (100–150 mg/day), and one patient was being treated with mycophenolate mofetil (1,000 mg/day). Twelve were on a maintenance dose of immunosuppressive drugs (prednisone: ≤10 mg/day, azathioprine: ≤100 mg/day). Patients with a previous history of pulmonary disorders other than SLE (asthma, chronic obstructive pulmonary disease, lung cancer, pneumonia or allergies) and with current infection were excluded from the study. The disease activity was evaluated by the systemic lupus activity measure (SLAM) (Griffiths et al. 2005) and the active disease was considered as SLAM >10. The active stage of SLE was found in six patients, including four persons treated with immunosuppressants (Table 1).

**Table 1:** Demographic and clinical characteristics of SLE patients and control group

	SLE patients (*n* = 34)	Control group (*n* = 31)
Age (years)	36.9 ± 11.8	31.2 ± 4.5
Gender		
Female	31 (91.2 %)	27 (87.1 %)
Male	3 (8.8 %)	4 (12.9 %)
Smoker	4 (11.8 %)	0 (0 %)
Disease duration (years)	8.9 ± 11.2	
SLAM score median (range)	8 (3–15)	
Active	6 (17.7 %)	
Inactive	28 (82.3 %)	
ANA ≥1:160	34 (100 %)	
P/MP therapy	16 (47 %)	
NSAID therapy	9 (26.5 %)	
Immunosuppressive (MM, A) therapy	4 (11.8 %)	

*A* azathioprine, *ANA* antinuclear antibody, *MM* mycophenolate mofetil, *MP* methylprednisolone, *NSAID* non-steroidal anti-inflammatory drug, *P* prednisone, *SLAM* systemic lupus activity measure

HRCT studies were performed using a 64-slice CT (GE Healthcare LightSpeed), with 1.25 mm single slices, collimation at 1-cm intervals through the lungs, 120 kV, 265 mA, and 0.6 s scan time. The images were obtained at a window level of −700 Hounsfield units (HU) and a window width of 1,500 HU and examined by two independent experienced radiologists.

Spirometry was performed according to European Respiratory Society (ERS) standards (Miller et al. 2005). The equipment (MES LUNGTEST 1000) was calibrated once daily. FVC and FEV_1_ were measured and the Tiffenau index was calculated.

Body plethysmography was obtained with a plethysmograph (Jaeger) and performed according to ERS standards (Wanger et al. 2005). Residual volume and total lung capacity (TLC) were measured.

DLCO diffusion capacity for carbon monoxide corrected for hemoglobin concentration was obtained with Lung Test 1000 Mes according to ATS/ERS guidelines (Macintyre et al. 2005).

EBC was collected by means of a condenser (Ecoscreen, Jaeger) and performed according to available recommendations (Horvath et al. 2005). After rinsing their mouth with distilled water, patients were asked to breathe out spontaneously for 10 min through a mouthpiece equipped with a saliva trap. A total volume of 1–3 ml of EBC samples was collected.

BALF was collected with a flexible bronchoscope (Olympus B1-IT20) according to British Thoracic Society guidelines (2001). The tip of the bronchoscope was wedged in the lingular segments and 5 aliquots of 50 ml of sterile 0.9 % NaCl solution at 37 °C were poured and recovered by gentle suction after each part. The fluid was collected, filtered through gauze and centrifuged. The pellet was suspended in phosphate buffer. Cytospins were prepared and the slides were stained with May-Grünwald Giemsa stain. The total cell count (TCC) was calculated (*n* × 10^6^) under a light microscope, and the numbers of macrophages, lymphocytes, neutrophils and eosinophils were presented as percentages of the TCC.

All samples of EBC and BALF were collected in the morning (8:00 and 10:00 a.m.) in order to avoid any influence of the circadian cycle and were immediately frozen at −80 °C until assayed. The IL-8 level in BALF and EBC samples was measured with a commercially available enzyme-immunosorbent assay kit (Quantikine, R&D Systems, USA) according to the manufacturer’s protocols (the sensitivity 3.4 pg/ml for IL-8). The assay employs the quantitative sandwich immunoassay technique.

### Statistical Analysis

The normality of distribution of continuous variables was tested using W test. The Student’s *t* test or Mann–Whitney’s *U* test was used for intergroup comparisons, depending on the distribution of respective variables. For comparisons of more than two groups, nonparametric analysis of variance with post hoc tests was performed using a Bonferroni-corrected Mann–Whitney’s *U* test for significant comparisons. Correlations were evaluated using the Pearson’s test or Spearman’s rank correlation test depending on normality of distribution. A *p* value of <0.05 was assumed to be statistically significant. The STATISTICA 10.0 (StatSoft, USA) package was used for analysis.

## Results

**Fig. 1:**
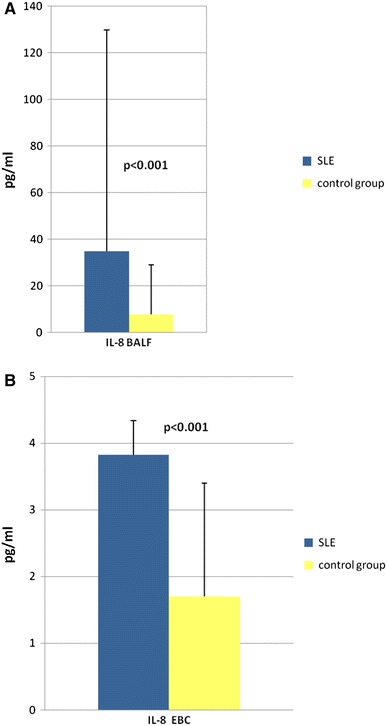
IL-8 concentrations in BALF and EBC in SLE patients compared to healthy controls

SLE patients had an increased percentage of neutrophils in BALF when compared with control group (1.00 ± 5.99 vs. 0.00 ± 0.56, *p* = 0.0003). The mean IL-8 concentrations in BALF and EBC were higher in SLE patients compared to healthy controls (34.84 ± 95.0 vs. 7.65 ± 21.22 pg/ml, *p* < 0.001; 3.82 ± 0.52 pg/m vs. 1.7 ± 1.7 pg/ml, *p* < 0.001, respectively; Fig. 1). The concentrations of IL-8 in the EBC of all healthy persons were below the detection limit and were arbitrarily assumed to be half of the detection limit value.

In the group of SLE patients, SLAM was correlated with the percentage of neutrophils, lymphocytes and macrophages in BALF (*r* = 0.44, *p* = 0.009; *r* = 0.54, *p* = 0.001; *r* = −0.52, *p* = 0.002, respectively). The activity of the disease correlated negatively with FEV_1_ and FVC (*r* = −0.50, *p* = 0.005; *r* = −0.57, *p* = 0.001, respectively; Table 2).

**Table 2:** Correlations of SLAM in SLE patients

Correlations	*r* value	*p* value
SLAM versus lymphocytes % BALF	0.54	0.001
SLAM versus macrophages % BALF	–0.52	0.002
SLAM versus neutrophils % BALF	0.44	0.009
SLAM versus eosinophils % BALF	–0.13	ns (*p* = 0.44)
SLAM versus IL-8 BALF	0.01	ns (*p* = 0.92)
SLAM versus IL-8 EBC	–0.28	ns (*p* = 0.10)
SLAM versus FEV_1_/FVC (%)	–0.03	ns (*p* = 0.86)
SLAM versus FEV_1_ (%pred.)	–0.50	0.005
SLAM versus FVC (%pred.)	–0.57	0.001
SLAM versus TLC (%pred.)	–0.31	ns (*p* = 0.15)

*BALF* bronchoalveolar lavage fluid, *EBC* exhaled breath condensate, *FEV*_*1*_ forced expiratory volume in 1 s, *FVC* forced vital capacity, *ns* not significant, *SLAM* systemic lupus activity measure, *TLC* total lung capacity, *%pred.* % predicted

**Fig. 2:**
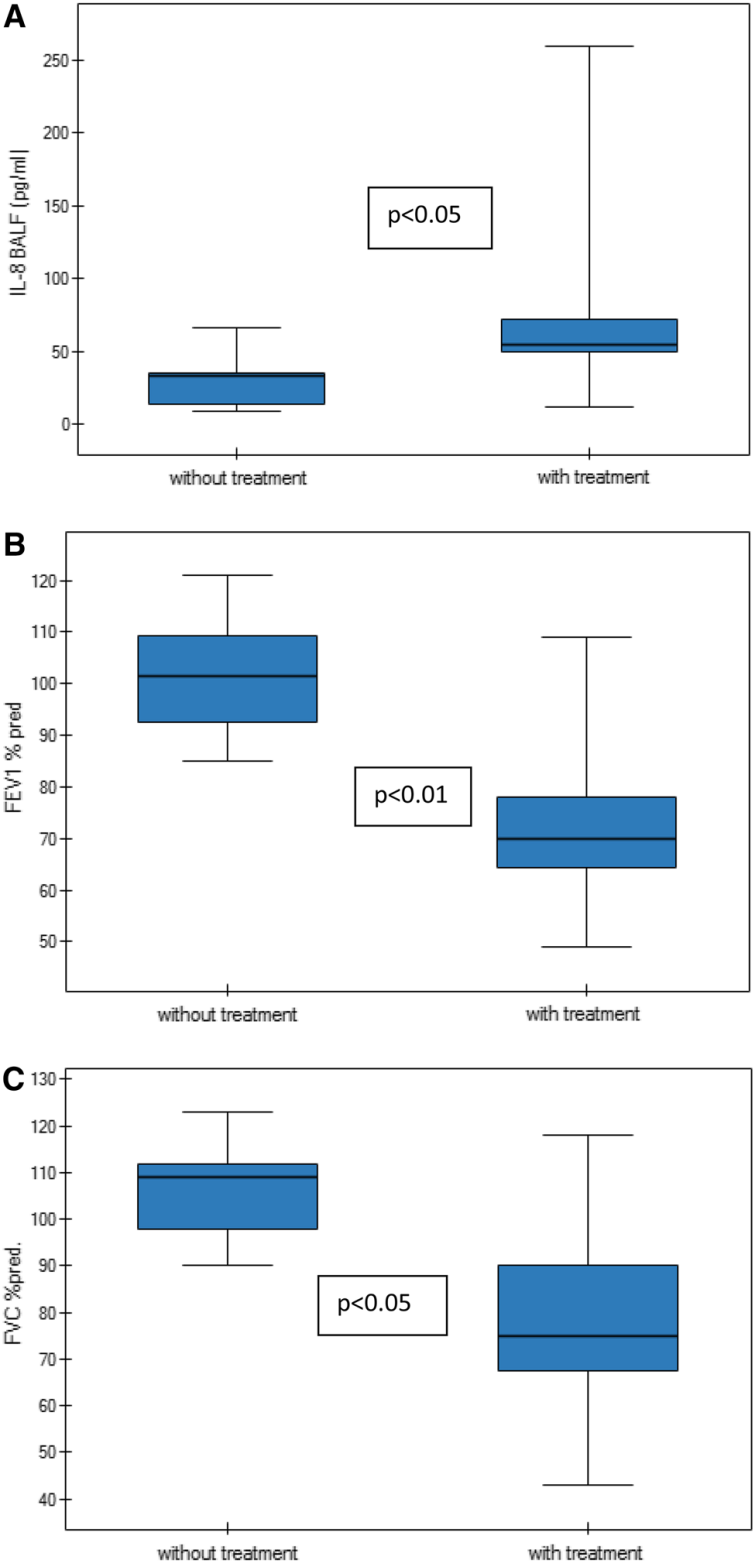
The comparison of IL-8 level in BALF, FEV_1_ and FVC of SLE patients with pulmonary fibrosis in relation to immunosuppressive treatment

**Fig. 3:**
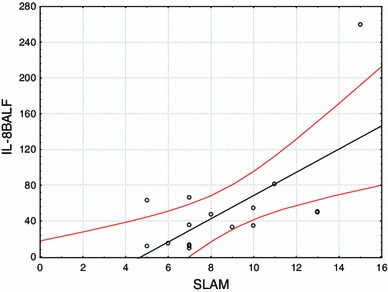
Positive correlation of SLAM with IL-8 concentration in BALF in SLE patients with pulmonary fibrosis (*r* = 0.65, *p* = 0.006)

Seventeen patients (50 %) were found to have pulmonary fibrosis in HRCT (Table 3). The disease activity scored by SLAM was significantly higher and TLC lower in the SLE patients with pulmonary fibrosis when compared with patients with normal HRCT (8.00 ± 3.17 vs. 6.00 ± 2.31; 88.00 ± 28.29 vs. 112.00 ± 21.08 % predicted, *p* = 0.01 in both). Demographic and clinical characteristics of SLE patients with and without pulmonary fibrosis are found in Table 4. The concentration of IL-8 in BALF and EBC, the spirometric results (FEV_1_/FVC, FEV_1_, FVC, DLCO) and the numbers of macrophages, lymphocytes, neutrophils and eosinophils in BALF were not significantly different in the patients with pulmonary fibrosis when compared with patients without pulmonary fibrosis (Table 5). Also there were no differences in the concentration of IL-8, the lung function tests or SLAM in SLE patients associated with immunosuppressive treatment (Table 6). However, among SLE patients with pulmonary fibrosis IL-8 concentration in BALF was significantly higher, FEV_1_ and FVC were significantly lower in patients with immunosuppressive treatment when compared with patients without immunosuppressive treatment (81.38 ± 81.23 vs. 29.61 ± 19.14, *p* < 0.05; 73.37 ± 18.67 vs. 101.75 ± 12.03, *p* < 0.01; 78.71 ± 23.76 vs. 106.00 ± 11.25, *p* < 0.05, respectively; Fig. 2; Table 7). SLAM was found to have negative correlations with FEV_1_ and FVC (*r* = −0.53, *p* = 0.035; *r* = −0.67, *p* = 0.006) and positive correlation with IL-8 concentration in BALF (*r* = 0.65, *p* = 0.006) in patients with pulmonary fibrosis (Fig. 3).

**Table 3:** HRCT positive radiological signs of pulmonary fibrosis in SLE patients

	SLE patients (*n* = 34)
HRCT changes	17 (50 %)
Reticular pattern	12 (35 %)
Areas of ground glass attenuation	2 (5.9 %)
Interlobular interstitial thickening	2 (5.9 %)
Air-space nodules	3 (8.8 %)
Honeycombing	2 (5.9 %)

**Table 4:** Demographic and clinical characteristics of SLE patients with and without pulmonary fibrosis

	SLE with pulmonary fibrosis (*n* = 17)	SLE without pulmonary fibrosis (*n* = 17)
Age (mean ± SD)	36.1 ± 11.2	37.2 ± 11.5
Smoker	2 (11.8 %)	2 (11.8 %)
Gender		
Female	16 (94.1 %)	15 (88.2 %)
Male	1 (5.9 %)	2 (11.8 %)
SLAM score median (range)	8.00 ± 3.17	6.00 ± 2.31
Active	5 (29.4 %)	1 (5.9 %)
Inactive	12 (70.6 %)	16 (94.1 %)
ANA ≥ 1:160	17 (100 %)	17 (100 %)
Positive anti-dsDNA	2 (11.8 %)	2 (11.8 %)
Anti-RNP	0 (0 %)	0 (0 %)
P/MP therapy	8 (47 %)	8 (47 %)
NSAID therapy	5 (29.4 %)	4 (23.5 %)
Immunosuppressive (MM, A) therapy	2 (11.8 %)	2 (11.8 %)
Fever	1 (5.9 %)	2 (11.8 %)
Skin and mucous membrane lesion	9 (52.9 %)	6 (35.3 %)
Arthritis	7 (41.2 %)	4 (23.5 %)
Renal active disorders	2 (11.8 %)	1 (5.9 %)
Cardiac disorders	1 (5.9 %)	0 (0 %)
Neuropsychiatric disorders	6 (35.3 %)	5 (29.4 %)
Gastrointestinal disorders	4 (23.5 %)	2 (11.8 %)
Lymphadenopathy	0 (0 %)	0 (0 %)
Anemia (Hb <12 g/dl)	3 (17.6 %)	3 (17.6)
Leucopenia (WBC <3.5 cc mm)	5 (29.4 %)	2 (11.8 %)
Thrombocytopenia (<150,000 cc mm)	2 (11.8 %)	1 (5.9 %)
ESR >25 mm/h	6 (35.3 %)	3 (17.6 %)

*A* azathioprine, *ANA* antinuclear antibody, *anti-ds DNA* anti-double stranded DNA, *anti-RNP* antibody to ribonucleoprotein, *ESR* erythrocyte sedimentation rate, *MM* mycophenolate mofetil, *MP* methylprednisolone, *NSAID* non-steroidal anti-inflammatory drug, *P* prednisone, *SLAM* systemic lupus activity measure

**Table 5:** The comparison of SLE patients with and without pulmonary fibrosis

Parameter	SLE with pulmonary fibrosis (*n* = 17)	SLE without pulmonary fibrosis (*n* = 17)	*p* value
SLAM	8.00 ± 3.17	6.00 ± 2.31	*p* = 0.01
Time of SLE duration (years)	11.00 ± 8.16	5.00 ± 6.33	ns (*p* = 0.42)
Lymphocytes % BALF	20.82 ± 12.27	16.00 ± 8.27	ns (*p* = 0.29)
Eosinophils % BALF	0.00 ± 1.94	1.00 ± 1.3	ns (*p* = 0.07)
Neutrophils % BALF	2.00 ± 8.02	1.00 ± 2.02	ns (*p* = 0.37)
Macrophages % BALF	81.00 ± 14.84	80.00 ± 8.04	ns (*p* = 0.19)
FEV_1_/FVC (%)	82.37 ± 8.14	85.50 ± 4.94	ns (*p* = 0.25)
FEV_1_ (%pred.)	90.50 ± 21.09	100.00 ± 16.59	ns (*p* = 0.09)
FVC (%pred.)	93.26 ± 22.44	106.00 ± 15.09	ns (*p* = 0.27)
TLC (%pred.)	88.00 ± 28.29	112.00 ± 21.08	*p* = 0.01
DLCOc (%pred.)	90.00 ± 28.32	98.00 ± 15.72	ns (*p* = 0.43)
IL-8 BALF (pg/ml)	41.3 ± 59.48	28.06 ± 121.23	ns (*p* = 0.42)
IL-8 EBC (pg/ml)	3.92 ± 0.39	3.78 ± 0.63	ns (*p* = 0.89)

Values are given as the mean ± SD

*BALF* bronchoalveolar lavage fluid, *DLCOc* diffusion capacity for carbon monoxide corrected for hemoglobin concentration, *EBC* exhaled breath condensate, *FEV*_1_ forced expiratory volume in 1 s, *FVC* forced vital capacity, *ns* not significant, *SLAM* systemic lupus activity measure, *TLC* total lung capacity, *%pred.* % predicted

**Table 6:** The comparison of SLE patients in relation to immunosuppressive treatment

Parameter	With immunosuppressive treatment (*n* = 16)	Without immunosuppressive treatment (*n* = 18)	*p* value
SLAM	8.00 ± 3.87	7.00 ± 1.90	ns (*p* = 0.35)
Time of SLE duration (years)	6.00 ± 6.27	8.00 ± 8.49	ns (*p* = 0.63)
Lymphocytes % BALF	16.00 ± 10.30	17.00 ± 10.10	ns (*p* = 0.79)
Eosinophils % BALF	0.00 ± 0.89	1.00 ± 2.09	ns (*p* = 0.052)
Neutrophils % BALF	2.50 ± 7.82	1.0 ± 3.77	ns (*p* = 0.41)
Macrophages % BALF	80.00 ± 11.13	82.00 ± 13.41	ns (*p* = 0.81)
FEV_1_/FVC (%)	82.20 ± 6.96	85.00 ± 6.69	ns (*p* = 0.29)
FEV_1_ (%pred.)	90.13 ± 25.25	100.00 ± 12.76	ns (*p* = 0.29)
FVC (%pred.)	97.50 ± 25.13	107.00 ± 12.73	ns (*p* = 0.53)
TLC (%pred.)	88.50 ± 36.35	97.00 ± 11.82	ns (*p* = 0.31)
DLCOc (%pred.)	99.00 ± 31.37	90.00 ± 13.38	ns (*p* = 0.14)
IL-8 BALF (pg/ml)	49.42 ± 135.00	29.63 ± 18.07	ns (*p* = 0.07)
IL-8 EBC (pg/ml)	3.82 ± 0.42	3.82 ± 0.63	ns (*p* = 0.64)

Values are given as the mean ± SD

*BALF* bronchoalveolar lavage fluid, *DLCOc* diffusion capacity for carbon monoxide corrected for hemoglobin concentration; *EBC* exhaled breath condensate, *FEV*_1_ forced expiratory volume in 1 s, *FVC* forced vital capacity, *ns* not significant, *SLAM* systemic lupus activity measure, *TLC* total lung capacity, *%pred.* % predicted

**Table 7:** The comparison of SLE patients with pulmonary fibrosis in relation to immunosuppressive treatment

Parameter	With immunosuppressive treatment (*n* = 16)	Without immunosuppressive treatment (*n* = 18)	*p* value
SLAM	10.75 ± 3.88	7.55 ± 1.23	ns
Time of SLE duration (years)	12.0 ± 6.48	8.57 ± 9.50	ns
Lymphocytes % BALF	18.50 ± 12.72	22.88 ± 12.23	ns
Eosinophils % BALF	1.44 ± 2.55	0.12 ± 0.35	ns
Neutrophils % BALF	7.37 ± 10.37	3.11 ± 4.98	ns
Macrophages % BALF	77.42 ± 14.19	72.22 ± 15.77	ns
FEV_1_/FVC (%)	79.12 ± 7.18	85.62 ± 8.15	ns
FVC (%pred.)	78.71 ± 23.76	106.00 ± 11.25	*p* < 0.05
FEV_1_ (%pred.)	73.37 ± 18.67	101.75 ± 12.03	*p* < 0.01
TLC (%pred.)	81.71 ± 16.34	97.50 ± 13.18	ns (*p* = 0.052)
DLCOc (%pred.)	98.87 ± 20.06	87.77 ± 13.85	ns
IL-8 BALF (pg/ml)	81.38 ± 81.23	29.61 ± 19.14	*p* < 0.05
IL-8 EBC (pg/ml)	3.76 ± 0.44	3.87 ± 0.38	ns

Values are given as the mean ± SD

*BALF* bronchoalveolar lavage fluid, *DLCOc* diffusion capacity for carbon monoxide corrected for hemoglobin concentration; *EBC* exhaled breath condensate, *FEV*_1_ forced expiratory volume in 1 s, *FVC* forced vital capacity, *ns* not significant, *SLAM* systemic lupus activity measure, *TLC* total lung capacity, *%pred.* % predicted

## Discussion

In the present study, the percentage of neutrophils in BALF was found to be higher in SLE patients when compared with healthy individuals and the findings are in accordance with previous research (BAL Cooperative Group Steering Committee 1990; Groen et al. 1993; Witt et al. 1996). Wallaert et al. (1990) reported lymphocytic predominance in the BAL fluid from lupus patients and impaired function of alveolar macrophages. They also proposed that subclinical alveolitis is present in SLE, with an accumulation of inflammatory and immune cells in the BAL fluid in patients without clinical and radiological respiratory abnormalities. These results indicate the presence of a cell-mediated immune response in the respiratory system of lupus patients.

Diffuse lung disease during lung biopsy and at autopsy has been observed in 4–70 % of SLE patients (Haupt et al. 1981). Interstitial lung disease is a relatively uncommon complication of SLE but the pulmonary changes attributable to interstitial abnormalities are found in CT scans of 30–38 % SLE patients with normal chest radiographs (Kakati et al. 2007; Keane and Lynch 2000). Additionally, HRCT reveals interstitial abnormalities in SLE patients with normal pulmonary function test results (Fenlon et al. 1996). Our results confirm a high occurrence of fibrotic changes in the lungs of SLE patients and the coexistence of fibrotic changes with normal lung function of lupus patients.

There is a growing body of evidence that chemokines are involved in the pathogenesis of diffuse lung disease. IL-8 is one of the major mediators of the inflammatory response and leads to neutrophil recruitment in the pulmonary interstitium and airspace. CXCL8 can activate a wide range of neutrophil functions including degranulation and respiratory burst (Knall et al. 1996). Sustained neutrophil accumulation in the alveolar space (neutrophilic alveolitis) causes lung injury and leads to interstitial fibrosis (Lynch et al. 1992; Pantelidis et al. 1997). Increased levels of IL-8 have been described in many inflammatory disorders characterized by neutrophilic infiltration, including psoriasis and rheumatoid arthritis (Biasi et al. 1998; Endo et al. 1991). The fact that IL-8 is a chemotactic cytokine which recruits the inflammatory cells to a particular location could make it a parameter of localized inflammation.

Al-Mutairi et al. (2007) report that lupus patients with pulmonary involvement have higher levels of pro-inflammatory cytokines in serum than those without pulmonary involvement. Lit et al. (2006) report a positive correlation of IL-8 plasma level with SLEDAI (systemic lupus erythematosus disease activity index in SLE patients). In the present study, the level of IL-8 was significantly higher in BALF and EBC of SLE patients. No significant differences were found in the numbers of particular BALF cells or IL-8 concentrations between patients with and without pulmonary fibrosis, despite the fact that SLAM correlates with IL-8 level in the BALF of patients with fibrotic changes in HRCT. We hypothesize that the lack of differences between patients with and without pulmonary fibrosis may be an effect of the influence of other factors like cytokines (tumor necrosis factor, IL-10), immunosuppressive therapy or the predominance of non-active SLE patients in the study. The immunosuppressive therapy is a limiting factor for the study accuracy.

An increased IL-8 level in BALF and serum of systemic sclerosis patients has been reported (Bolster et al. 1997; Furuse et al. 2003; Meloni et al. 2004). Moreover, Furuse et al. (2003) detected that elevated serum IL-8 concentrations significantly correlated with decreased percentage of DLCO in systemic sclerosis patients. They suggested that the elevation of chemokines correlates with pulmonary involvements.

The level of IL-8 mRNA in the idiopathic pulmonary fibrosis (IPF) patients correlated with the number of neutrophils in BALF and with the degree of disease severity (Carre et al. 1991). Southcott et al. (1995) suggested that the levels of IL-8 in IPF may correlate with poor prognosis.

In our study SLE patients with pulmonary fibrosis and immunosuppressive treatment had higher IL-8 concentration in BALF when compared with patients with pulmonary fibrosis and without immunosuppressive treatment. Thomas et al. (2002) observed that steroids (dexamethasone) inhibited IL-8 secretion from peripheral blood mononuclear cells, but on the other hand the inhibitory effects of steroids on IL-8 secretion were absent in infants and they developed more severe bronchiolitis. In the present study, the immunosuppressive therapy altered the results and is a limiting factor for the study. The inhibitory effects of steroids on IL-8 secretion in SLE need to be studied further.

In conclusion, to the best of our knowledge, this is the first study estimating the concentration of IL-8 in the EBC and BALF of SLE patients with and without pulmonary fibrosis. Our observations confirm the importance of IL-8 in the pathogenesis of SLE. The increased concentration of IL-8 in BALF could be considered as a marker of pulmonary fibrosis in SLE. The decision whether to start treatment is often the most difficult clinical challenge, because many patients have limited pulmonary fibrosis that will not necessarily progress. Immunosuppressive drugs could be administered to patients with an increased level of IL-8 in BALF. IL-8 in BALF may be a valuable biomarker to monitor disease activity in SLE patients with pulmonary fibrosis. IL-8 could also be used as a potential therapeutic target of pulmonary fibrosis in SLE. Further studies are necessary for the evaluation of the prognostic role of these biomarkers in the EBC and BALF of SLE patients and the inhibitory effects of steroids on IL-8 secretion in SLE patients.
